# Plasma Protein Profiling to Discern Indolent from Advanced Systemic Mastocytosis

**DOI:** 10.1016/j.jmoldx.2024.05.010

**Published:** 2024-06-24

**Authors:** Cristina Iribarren, Kerstin H. Levedahl, Ionut Atanasoai, Mattias Mattsson, Martin Höglund, Stina Söderlund, Hans Hägglund, Niclas Eriksson, Marie Carlson, Gunnar P. Nilsson

**Affiliations:** ∗ Division of Immunology and Allergy, Department of Medicine Solna, and Center for Molecular Medicine, Karolinska Institutet, Karolinska University Hospital, Stockholm, Sweden; † Department of Public Health and Caring Sciences, Uppsala University, Uppsala, Sweden; ‡ Section of Hematology, Department of Medical Sciences, Uppsala University, Uppsala, Sweden; § Department of Immunology Genetics and Pathology, Uppsala University, Uppsala, Sweden; ¶ Uppsala Clinical Research Center, Uppsala University, Uppsala, Sweden; ‖ Gastroenterology Research Group, Department of Medical Sciences, Uppsala University, Uppsala, Sweden

## Abstract

Mastocytosis is a heterogeneous disorder characterized by abnormal mast cell accumulation, in which the clinical severity may be explained by distinct molecular mechanisms. This study aimed to explore plasma protein biomarkers associated with systemic mastocytosis subtypes, as well as the cellular origin of the identified proteins. Plasma samples from patients with mastocytosis, including cutaneous mastocytosis (CM), indolent systemic mastocytosis (ISM), and advanced systemic mastocytosis (AdvSM), and a reference group of patients with polycythemia vera, were analyzed by Proximity Extension Assay technology targeting 275 proteins. Furthermore, potential cellular origin was explored using an available single-cell RNA-sequencing data set generated from patients with ISM. The study cohort included 16 patients with CM, 92 patients with systemic mastocytosis (ISM, *n* = 80; AdvSM, *n* = 12), and 60 patients with polycythemia vera. A principal component analysis based on 275 plasma proteins revealed one cluster of patients with CM and ISM that was separated from patients with AdvSM. Up to 29 proteins were associated with distinct severe activity in patients with systemic mastocytosis (ISM versus AdvSM), including IL-1 receptor type 1 (IL-1RT1) and tumor necrosis factor ligand superfamily member 13B (TNFSF13B) (q < 0.01). Furthermore, single-cell RNA-sequencing analysis from ISM-derived bone marrow cells revealed that the mRNA for the identified proteins was not exclusive of mast cells. Distinct plasma protein profiles show potential to refine ISM and AdvSM diagnoses, possibly reflecting differences in pathogenic mechanisms and diverse clinical manifestations.

## Graphical abstract

**Figure:**
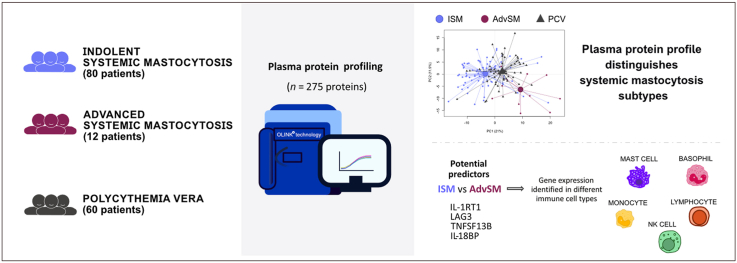

Mastocytosis is a myeloproliferative disorder characterized by an increase in abnormal, clonal, mast cells. The disease is classified into cutaneous mastocytosis (CM), affecting only the skin; and systemic mastocytosis (SM), where at least one extracutaneous organ is involved. SM is further divided into indolent SM (ISM), smoldering SM, bone marrow mastocytosis, aggressive SM, SM with an associated hematological neoplasm (SM-AHN), and mast cell leukemia.^1^ Aggressive SM, SM-AHN, and mast cell leukemia account for approximately 10% of the patients with SM and are often referred to as advanced SM (AdvSM) with worse prognosis. The symptoms derive from increased release of mast cell mediators and/or organ failure attributable to mast cell infiltration. In particular, the wide range of clinical manifestations of SM, from indolent and good prognosis to organ impairment and often fatal outcome in AdvSM, entangles patient diagnosis and accentuates the need of understanding its complex pathophysiology.^2^

Several studies have previously made search attempts for new evidence of immune mediators and cell types involved in mastocytosis pathogenesis. At diagnosis, most patients with mastocytosis display increased levels of mast cell mediators, such as serum tryptase,^3^ which also is one of the minor criteria for the diagnosis of SM.^1^ Other mast cell mediators that can be increased and detected in the urine include metabolites of histamine^4^ and prostaglandin D_2_.^5^^,^^6^ In subtypes of SM, recent studies have identified high protein expression of certain proinflammatory cytokines and chemokines^7^, ^8^, ^9^, ^10^, ^11^ and imbalance of immune cell subpopulations^8^, ^9^, ^10^^,^^12^^,^^13^ in peripheral blood. In some cases, the forementioned markers correlated with symptom-specific cases,^12^^,^^14^, ^15^, ^16^ severity,^8^^,^^17^ and disease extension or category,^11^^,^^14^^,^^18^^,^^19^ whereas the prediction of phenotypes has had little reward. Therefore, although only tryptase contributes to the current diagnostic criteria, it is not pathognomonic and may harbor another myeloid hematologic disorder.^20^

Polycythemia vera (PCV) is a myeloproliferative disorder characterized by overproduction of clonal *JAK2*-mutated erythrocytes in the bone marrow^20^^,^^21^ and chronic inflammation.^22^^,^^23^ Interestingly, PCV can be associated with SM,^24^ where mast cell mediators seem to contribute to pruritus pathogenesis.^25^^,^^26^ However, PCV is per se not a mast cell disorder.

Considering the evident heterogeneity among patients with mastocytosis, there is a need to broaden the focus of molecular components that may contribute to the pathogenesis of this disorder. We hypothesized that the clustering of patients based on proteomic data could improve diagnosis and give insight to disease mechanisms underlying a clinically diverse disease, such as mastocytosis. Therefore, this study aimed to identify plasma biomarkers associated with specific mastocytosis subtypes using proteomic analysis, as well as the cellular origin of the identified proteins. The differences in the pathophysiological mechanisms underlying SM and PCV were also addressed.

## Materials and Methods

### Study Cohorts

Plasma samples were obtained from the Uppsala Umeå Comprehensive Cancer Consortium biobank.^27^ Adult patients with mastocytosis (*n* = 108) were included from the Centre of Excellence in Mastocytosis at Uppsala University Hospital (Uppsala, Sweden). Patients were diagnosed after a comprehensive medical evaluation following the 2016 World Health Organization mastocytosis classification,^21^^,^^28^ which included a bone marrow examination in all patients. Patients were then categorized as having mastocytosis restricted to the skin (CM) or with systemic involvement (patients with SM). Patients with SM were further characterized on the basis of clinical aggressiveness into ISM, including three patients with smoldering SM (Supplemental Table S1), and AdvSM. The AdvSM group included patients with either SM-AHN or aggressive SM. At the sampling time, some of the patients followed treatment for their mediator-related symptoms (eg, with H1 and/or H2 receptor antagonists). Two patients, one with aggressive SM-AHN and another with ISM, were under interferon treatment at the time of sampling; one patient with SM-AHN followed hydroxyurea treatment, and one last patient with ISM was receiving psoralen plus UVA therapy. None of the included patients with SM were on treatment with kinase inhibitors or cytoreductive agents during sample collection. In addition, 60 adult patients with PCV were included. The diagnosis was made according to the 2008 World Health Organization diagnostic classification for myeloproliferative neoplasm.^21^^,^^29^ All included patients provided samples and written informed consent. This study was approved by the Regional Ethical Review Board in Uppsala (Dnr. 2010/198/5) and the Swedish Ethical Review Authority (Dnr. 2019-02496) and conducted following the Declaration of Helsinki.

### Tryptase Analysis

Tryptase was assayed in serum at the Academic Laboratory, Department of Clinical Immunology and Transfusion Medicine, University Hospital (Uppsala, Sweden). The reference value was <11.4 μg/L.

### Proteomic Analysis

The protein profile was analyzed in plasma samples by Proximity Extension Assay technology (Olink Proteomics, Uppsala, Sweden) at the Clinical Biomarker Facility, SciLifeLab (Uppsala, Sweden). For details about the method, see the study by Assarsson et al.^30^ The targeted profile included 276 proteins from the following panels: Olink Target 96 Cardiovascular II version 5006 (Supplemental Table S2), Olink Target 96 Cardiovascular III version 6113 (Supplemental Table S3), and Olink Target 96 Immune Response version 3204 (Supplemental Table S4). Relative protein concentration was expressed as NPX (log_2_ normalized protein expression). Of the analyzed profile, one sample from the PCV group failed the Cardiovascular II panel and five samples did not pass the quality control (PCV: *n* = 1; ISM: *n* = 2; AdvSM: *n* = 2) but were kept in the analysis. Similarly, analyzed proteins with low detection levels were included as a conservative measure. These proteins were marked with a double dagger symbol (‡) when having >20% of the values below the limit of detection and should be interpreted cautiously. Because IL-6 was part of both the Cardiovascular II and the Immune Response panels, the duplicate detected below the limit of detection in a greater percentage of samples was excluded (ie, IL-6 from the Immune Response panel). As a consequence, the proteomic profile consisted of 275 detected proteins, and all samples (*n* = 168) were included in the final statistical analyses.

### Statistical Analysis

#### Univariate Analysis

The study included all subjects who had been referred to the Uppsala University Hospital Center of Excellence in Mastocytosis, and biobanked in the Uppsala Umeå Comprehensive Cancer Consortium biobank, up to the date of the start of the study, and no power calculation was perfomed. Demographic characteristics at inclusion were analyzed using the SPSS Statistical Package (released 2021; IBM SPSS Statistics for Windows version 28.0; IBM Corp., Armonk, NY). The association between categorical variables was determined with the χ^2^ test of independence, whereas differences of continuous variables between groups were determined by Kruskal-Wallis H test. Biological data were analyzed using the stats package in R version 4.2.2 (R Foundation for Statistical Computing, Vienna, Austria, *https://www.r-project.org*). Specific NPX levels were compared between groups using the Welch two-sample *t*-test with false discovery rate correction. All between-group comparisons were performed using a parametric approach assuming unequal variances attributable to differences of sample size. The data are shown as frequency and percentage or mean (SD), depending on the test used, and *P* < 0.05 and false discovery rate–adjusted *P* (q) < 0.05 were interpreted as statistically significant.

#### Principal Component Analysis

The plasma protein profile was assessed to explore clustering of patients using unsupervised analysis and R version 4.2.2. Missing values were first estimated using the imputePCA function (missMDA package). Principal component analysis (PCA) plots were computed using the prcomp function on scaled data and visualized using an in-house script with similar outcome as the pca2d function (pca3d package unavailable in CRAN from February 2023). The centroids represented the normal arithmetic means of the groups. The permutation of the PCA analysis clusters was performed using an in-house script published elsewhere.^31^ The statistical significance of the distance between centroids was evaluated by ranking the actual centroid difference (cluster A – cluster B) within a simulated null distribution established from iterative randomization of the group labels. *P* < 0.05 was interpreted as significantly different group means.

#### Volcano Plot

Differentiating protein biomarkers were visualized in volcano plots generated using the ggplot2 and ggrepel packages in R. These plots were generated motivated by the olink_volcano_plot function of the OlinkAnalyze version 3.2.2 package in R (*https://cran.r-project.org/web/packages/OlinkAnalyze/index.html*). The difference in mean NPX between groups (estimate, *x* axis) was calculated, and tested by the Welch two-sample *t*-test and false discovery rate method [–log_10_(q), *y* axis] from the stats package in R (*https://cran.r-project.org/web/packages/STAT/index.html*). Because NPX is on the log_2_ scale, the difference in NPX between two groups would essentially be the same as the log_2_ fold change. Dots were colored based on q value cutoff of 0.05 and the corresponding study group; protein names are annotated if relevant.

#### Feature Selection

The Boruta algorithm was used as the feature selection method to reduce the dimensionality of the data and enable identification of the most relevant (weakly or strongly) discriminative proteins.^32^ First, lm function (stats package in R) was used to adjust protein values for age and sex, and missing values were replaced using na.roughfix function (randomForest package).^33^ Boruta was run in the resulting data frame (ie, signal). In short, Boruta algorithm (based on random forest) uses the original data frame (signal) and the shuffled version of these data (null hypothesis, equivalent to shadow attributes or random noise). All variables compete in the prediction of the diagnosis label through multiple random forests that compare the Z-scores_SIGNAL_ with the Z-score_ATTRIBUTE_. As a result, variables that perform better than the random noise (higher Z-score) are interpreted as confirmed variables, those that perform worse (lower Z-score) are the rejected variables, and variables that cannot be classified are labeled as tentative. Importance was calculated as normalized permutation importance (getImpRfZ). The algorithm was run using the Boruta package,^32^ set.seed(9999), doTrace = 2 (verbosity level), and maxRuns = 500. Importance was calculated as normalized permutation importance (getImpRfZ). The mean abundance of confirmed variables within each study group and in relation to the reported gastrointestinal symptoms was depicted in a Cleveland plot using the ggplot2, dplyr, and tidyr packages in R.

#### Binary Logistic Regression

The relationships between top confirmed variables [x = predictors with the greatest importance (Z-score) values] and outcome (y = diagnosis) were explored using binary logistic regression. All models were adjusted to age and conducted using the lrm function (rms package) in R. Odds ratio was calculated by exp(β) and represents the odds per increase of one unit in a specific marker. The corresponding 95% CI was calculated as follows: β ± 1.96 × SEM. Last, lrtest function (likelihood ratio) tested the overall potential contribution of adding more markers to the model. *P* < 0.05 was considered statistically significant.

### Analysis of scRNA-Seq Data

Gene expression and cellular origin of the top proteins were investigated in a single-cell RNA-sequencing (scRNA-seq) data set published elsewhere.^8^ This scRNA-seq data set file contained normalized and log-transformed gene expression data generated from bone marrow mononuclear cells of three patients with ISM. The data set is available at the Gene Expression Omnibus database^8^ (*https://www.ncbi.nlm.nih.gov/geo*; accession number GSE222830), and cell type annotation was refined on the basis of the study by Rosell et al.^34^ Analyses and visualizations were done in R version 4.3.1. The dotPlot function (scater package) was used to compute the average gene expression and the percentage of cells that express each gene within a specific cell type, and ggplot2 version 3.4.3 was used for visualization. Gene-wise standardization of the average gene expression across groups was used to highlight cell type specificities. Gene expression was mapped onto the available uniform manifold approximation and projection coordinates using ggplot2.

## Results

### Demographics and Clinical Characteristics

**Figure 1:**
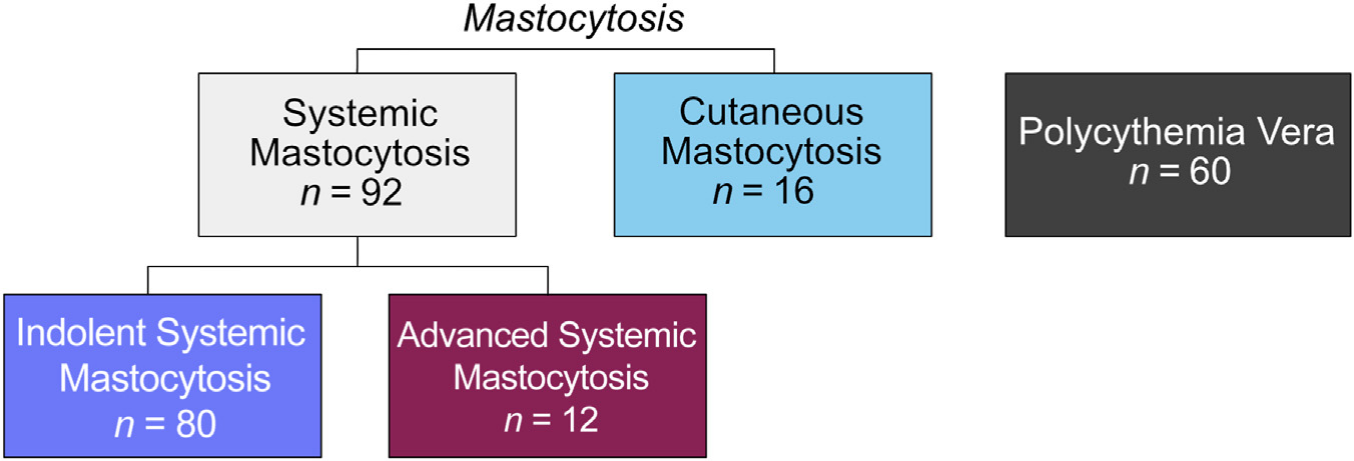
Overview of the patient groups included in the study.

This exploratory study included patients with CM (*n* = 16), ISM (*n* = 80), and AdvSM (*n* = 12), and 60 patients with PCV who constituted the reference group (Figure 1). Concerning demographic characteristics, sex proportion was evenly distributed between all study groups, whereas patients with PCV and AdvSM were older than patients with CM and ISM (*P* < 0.001) (Table 1). Patients with AdvSM had higher levels of serum tryptase than patients with CM and ISM (*P* < 0.01) (Table 1). For additional information on patients' characteristics and symptoms, see Table 1; and for detailed diagnosis, see Supplemental Table S1.

**Table 1:** Demographics and Clinical Characteristics of the Study Groups

Variable	CM (*n* = 16)	ISM (*n* = 80)	AdvSM (*n* = 12)	PCV (*n* = 60)	*P* value
Sex, F:M∗	9:7	54:26	5:7	39:21	0.33
Age, years†	45 (24–78)	59 (25–89)	72 (49–76)	72 (34–90)	<0.001
Altered BMMC morphology∗‡	0	67	12	–	–
CD2/CD25∗‡	1	73	12	–	–
BM c-kit mutation∗‡	10	67	11	–	–
Mutations different than c-kit∗‡					
*JAK2*	1	0	4	60	–
*TET2*	0	1	4	0	–
*ASXL1*	0	1	2	0	–
*SRSF2*	0	1	3	0	–
Other mutation	0	3	3	0	–
Skin symptoms∗‡	13	63	8	–	–
Anaphylaxis∗‡	1	22	1	0	–
Allergen-specific IgE antigen∗‡	2	23	0	0	–
Tryptase, ng/mL§	16 (8–39)	26 (20–49)	60.5 (30–135.5)	–	0.009

–, Not applicable; F, female; M, male; AdvSM, advanced systemic mastocytosis; BM, bone marrow; BMMC, BM mast cell; CM, cutaneous mastocytosis; ISM, indolent systemic mastocytosis; PCV, polycythemia vera.

∗ Frequency of patients.

† Median (range, minimum to maximum).

‡ Frequencies based on available data and positive answer. No statistical tests are applicable.

§ Median (interquartile range). Association between categorical variables was determined by χ^2^ test of independence. Between-group differences in continuous variables were determined by Kruskal-Wallis H test.

#### Altered Plasma Protein Profile in Patients with Indolent Compared with Advanced Systemic Mastocytosis

**Figure 2:**
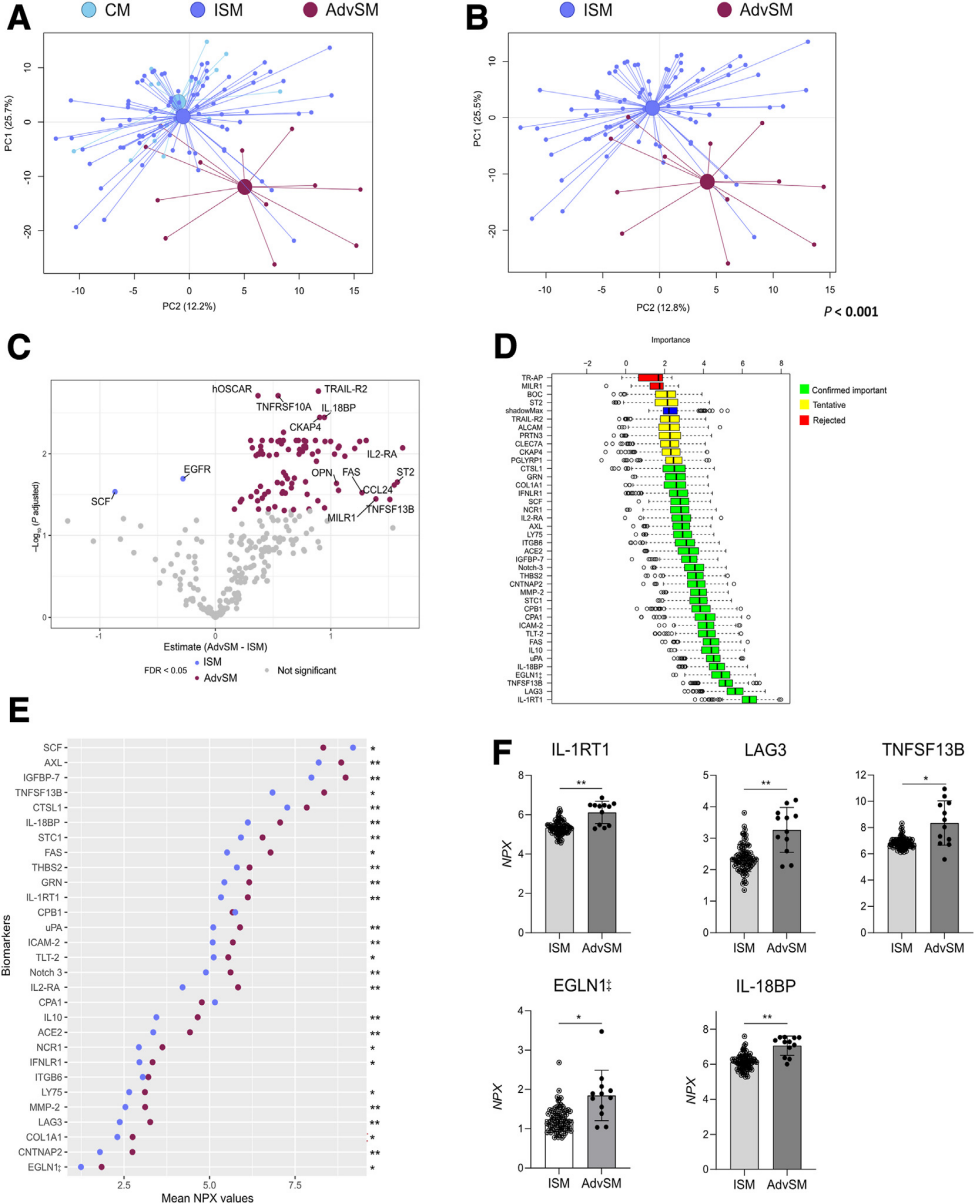
The protein profile of plasma samples of patients with mastocytosis presenting with different clinical disease severity and prognosis. Plasma proteins were analyzed using Olink technology. **A** and **B:** Principal component (PC) analysis score scatterplot based on the plasma protein profile showing patients with cutaneous mastocytosis (CM; cyan), indolent systemic mastocytosis (ISM; light blue), and advanced systemic mastocytosis (AdvSM; dark red; **A**) and patients diagnosed with the main subtypes of systemic mastocytosis (**B**). **A** and **B:** The centroids depict the group weighted average, and the *P* value supports the distance between the study groups' centroids. **C:** Volcano plot showing estimate mean difference versus false discovery rate (FDR)–adjusted *P* value (q; shown as log_10_) for each plasma protein in patients with AdvSM with respect to ISM. Each dot corresponds to the difference in mean NPX [log_2_ normalized expression of a biomarker between the study groups (AdvSM – ISM)]. When q < 0.05, biomarkers with increased levels in ISM are colored in light blue, whereas increased levels of biomarkers in AdvSM are colored in dark red. Nonsignificant biomarkers are colored in light gray. **D:** Variable importance chart using the Boruta algorithm showing the top 39 features of the plasma protein profile in ISM with respect to AdvSM. Green, yellow, and red box plots indicate the minimal, average, and maximum Z-score of confirmed important, tentative, and rejected attributes, respectively. Blue box plot represents Z-score of a shadow attribute. **E:** Cleveland dot plot illustrating the mean NPX values of 29 important biomarkers in ISM and AdvSM identified in the Boruta analysis. Mean values of the individual plasma biomarkers are depicted for each study group, and **asterisks** indicate statistical significance between the groups. **F:** Scatterplot with bar showing the plasma levels (NPX) of the top five biomarkers most relevant computed by the Boruta algorithm. **C**, **E**, and **F:** Welch two-sample *t*-test with FDR correction was used to identify differences between the groups. ^‡^More than 20% of samples below the limit of detection (LOD). *n* = 275 detected proteins (**A** and **B**); *n* = 16 patients with CM (**A**); *n* = 80 patients with ISM (**A**); *n* = 12 patients with AdvSM (**A**); *n* = 2 biomarkers with increased levels in ISM (**C**); *n* = 85 biomarkers with increased levels in AdvSM (**C**). ∗q < 0.05, ∗∗q < 0.01. EGLN1, Egl nine homolog 1; IL-1RT1, IL-1 receptor type 1; IL-18BP, IL-18-binding protein; LAG3, lymphocyte activation gene 3; TNFSF13B, tumor necrosis factor ligand superfamily member 13B.

The plasma protein profile of the 275 analyzed proteins differed between patients with mastocytosis on the basis of the clinical severity and prognosis. In a PCA based on the plasma protein profile, the clusters of patients with CM and ISM were clearly separated from the patient group with AdvSM (Figure 2A). The distance between the centroids of ISM and AdvSM subgroups was confirmed in a second PCA (*P* < 0.001) (Figure 2B). The levels of 85 proteins, including TNFSF13B, IL-18 binding protein (IL-18BP), and IL-2 receptor subunit alpha (IL2-RA; CD25), were increased, whereas levels of stem cell factor (SCF) and epidermal growth factor receptor (EGFR) were lower in patients with AdvSM (versus ISM), as depicted in a volcano plot (Figure 2C). The Boruta algorithm identified 29 confirmed biomarkers to be important for distinction of ISM with respect to AdvSM (Figure 2D). Of these biomarkers, most were detected in higher levels in plasma samples from patients with AdvSM over patients with ISM (Figure 2E), including the top five proteins: IL-1 receptor type 1 (IL-1RT1), lymphocyte activation gene 3 (LAG3), TNFSF13B (B-cell activating factor), Egl nine homolog 1 (EGLN1‡), and IL-18BP (Figure 2F).

#### Distinct Plasma Protein Profile Between Patients with Systemic Mastocytosis and Polycythemia Vera

**Figure 3:**
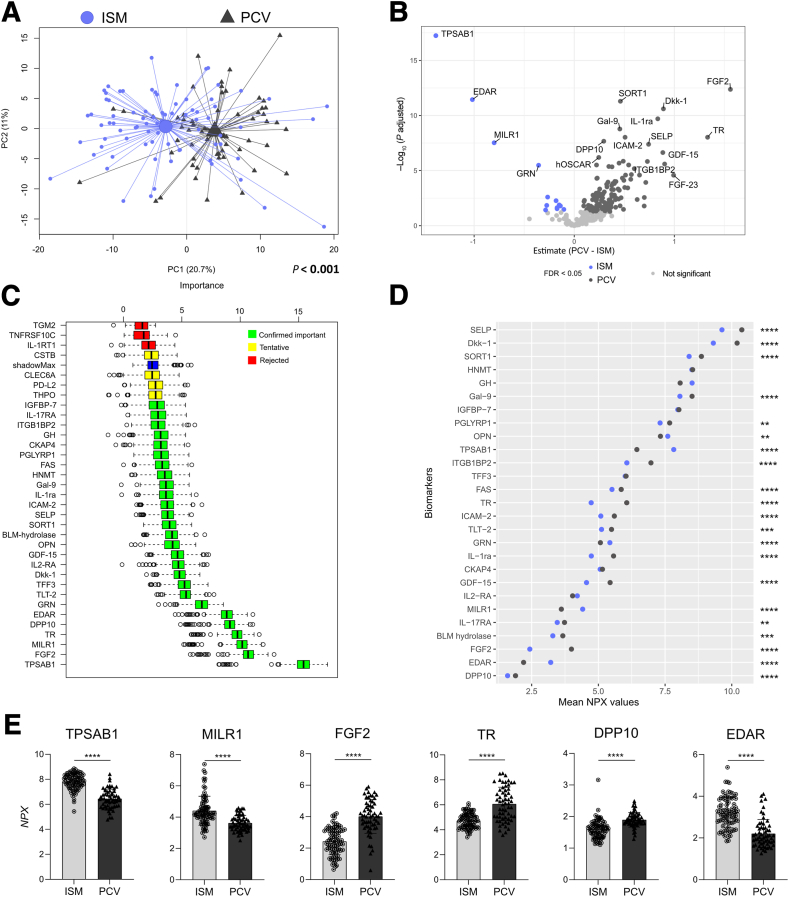
The protein profile of plasma samples of patients with indolent systemic mastocytosis (ISM) and polycythemia vera (PCV). Plasma proteins were analyzed using Olink technology. **A:** Principal component (PC) analysis score scatterplot based on the plasma protein profile showing patients with ISM (light blue circles) and PCV (dark gray triangles). The centroids depict the group weighted average, and the *P* value supports the distance between the study group's centroids. **B:** Volcano plot showing estimate mean NPX (log_2_ normalized protein expression) difference versus false discovery rate (FDR)–adjusted *P* value (q; shown as log_10_) adjusted statistical significance for each plasma protein in patients with PCV with respect to ISM. Each dot corresponds to the difference in mean NPX of a biomarker between the study groups (PCV – ISM). When q < 0.05, biomarkers with increased levels in ISM are colored in purple, and increased levels of biomarkers in PCV are colored in dark gray. Nonsignificant biomarkers are colored in light gray. **C:** Variable importance chart using the Boruta algorithm showing the top 34 features of the plasma protein profile in ISM with respect to PCV. Green, yellow, and red box plots indicate the minimal, average, and maximum Z-score of confirmed important, tentative, and rejected attributes, respectively. Blue box plot represents Z-score of a shadow attribute. **D:** Cleveland dot plot illustrating the 27 important biomarkers in ISM and PCV identified in the Boruta analysis. Mean values of the individual plasma biomarkers are depicted for each study group, and **asterisks** indicate statistical significance between the groups. **E:** Scatterplot with bar showing the plasma levels (NPX) of the top five biomarkers most relevant computed by the Boruta algorithm. **B**, **D**, and **E:** Welch two-sample *t*-test with FDR correction was used to identify differences between the groups. *n* = 275 detected proteins (**A**); *n* = 80 patients with ISM (**A**); *n* = 60 patients with PCV (**A**); *n* = 13 biomarkers with increased levels in ISM (**B**); *n* = 134 biomarkers with increased levels in PCV (**B**). ∗∗q < 0.01, ∗∗∗q < 0.001, and ∗∗∗∗q < 0.0001. DPP10, inactive dipeptidyl peptidase 10; EDAR, tumor necrosis factor receptor superfamily member EDAR; FGF2, fibroblast growth factor 2; MILR1, allergin-1; TPSAB1, tryptase alpha/beta-1; TR, transferrin receptor 1.

Next, to examine that the plasma protein profiles of patients with SM are not related to a hematologic neoplasm per se, the profiles of patients with ISM and AdvSM were compared with that of patients with PCV, a non–mast cell–dependent myeloproliferative neoplasm. The overall protein profile tended to differ between the main study groups despite the overlap of some patients with the different diagnosis (*P* < 0.001) (Supplemental Figure S1). Supported by a PCA, patients with ISM presented with a distinct protein profile that separated from patients with PCV (*P* < 0.001) despite the overlap (Figure 3A). A volcano plot depicted increased levels of 13 protein biomarkers, such as tryptase alpha/beta-1 (TPSAB1), tumor necrosis factor receptor superfamily member EDAR (EDAR), allergin-1 (MILR1), and granulin (GRN), in patients with ISM, whereas high levels of 134 biomarkers, including fibroblast growth factor 2 (FGF2) and transferrin receptor 1 (TR), were found in patients with PCV (Figure 3B). Using the Boruta algorithm, the distinction of ISM and PCV was defined by 27 confirmed protein biomarkers, where TPSAB1 was found to be the most important feature (Figure 3C) despite not being found with the highest NPX levels (Figure 3D). Most of the relevant biomarkers in the model were detected in low levels in patients with ISM versus PCV (Figure 3D), including FGF2 and TR (Figure 3E).

**Figure 4:**
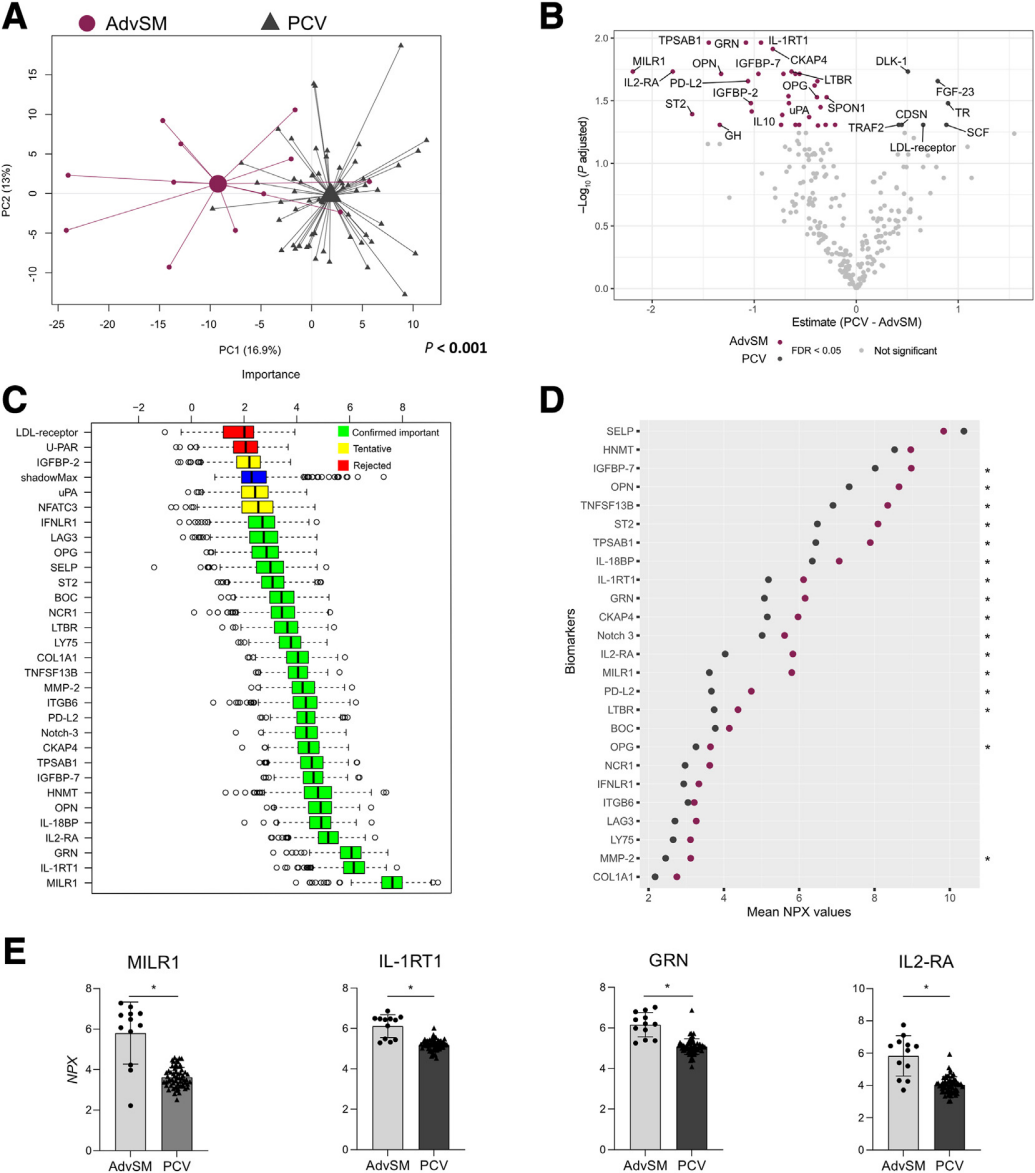
The protein profile of plasma samples of patients with advanced systemic mastocytosis (AdvSM) and polycythemia vera (PCV). Plasma proteins were analyzed using Olink technology. **A:** Principal component (PC) analysis score scatterplot based on the plasma protein profile showing patients with AdvSM (dark red circles) and PCV (dark gray triangles). The centroids depict the group weighted average, and the *P* value supports the distance between the study group's centroids. **B:** Volcano plot showing estimate mean NPX (log_2_ normalized protein expression) difference versus false discovery rate (FDR)–adjusted *P* value (q; shown as log_10_) adjusted statistical significance for each plasma protein in patients with PCV with respect to AdvSM. Each dot corresponds to the difference in mean NPX of a biomarker between the study groups (PCV – AdvSM). When q < 0.05, biomarkers with increased levels in AdvSM are colored in dark red, and increased levels of biomarkers in PCV are colored in dark gray. Nonsignificant biomarkers are colored in light gray. **C:** Variable importance chart using the Boruta algorithm showing the top 30 features for the AdvSM and PCV groups. Green, yellow, and red box plots indicate the minimal, average, and maximum Z-score of confirmed important, tentative, and rejected attributes, respectively. Blue box plot represents Z-score of a shadow attribute. **D:** Cleveland dot plot illustrating the 25 important biomarkers in AdvSM and PCV identified in the Boruta analysis. Mean values of the individual plasma biomarkers are depicted for each study group, and **asterisks** indicate statistical significance between the groups. **E:** Scatterplot with bar showing the plasma levels (NPX) of the top five biomarkers most relevant computed by the Boruta algorithm. **B**, **D**, and **E:** Welch two-sample *t*-test with FDR correction was used to identify differences between the groups. *n* = 275 detected proteins (**A**); *n* = 12 patients with AdvSM (**A**); *n* = 60 patients with PCV (**A**); *n* = 32 biomarkers with increased levels in AdvSM (**B**); *n* = 7 biomarkers with increased levels in PCV (**B**). ∗q < 0.05. GRN, granulin; IL-1RT1, IL-1 receptor type 1; IL2-RA, IL-2 receptor subunit alpha; MILR1, allergin-1.

Similarly, the plasma protein profile was altered in patients with AdvSM versus PCV (*P* < 0.001) (Figure 4A). A volcano plot depicted a great number of altered biomarkers in AdvSM (*n* = 32 versus 7 protein biomarkers) (Figure 4B). Of the complete proteomic profile, high NPX levels of 24 biomarkers were confirmed to be relevant for the distinction of AdvSM and PCV (Figure 4, C and D), where MILR1 was identified as the protein leading the distinction (Figure 4E).

#### Identification of Potential Predictors of Systemic Mastocytosis Subtypes

To further explore if a set of the top biomarkers could predict ISM versus AdvSM, a binary logistic regression was applied. Step-by-step addition of markers was also conducted to evaluate whether it improved the corresponding prediction model (Table 2). Age-adjusted protein levels of IL-1RT1, LAG3, TNFSF13B, EGLN‡, or IL-18BP were identified as independent predictors delineating patients with ISM diagnosis versus AdvSM, although the individual odds ratios were low (odds ratio ≤ 0.21; *P* ≤ 0.01). The gradual addition of the forementioned biomarkers confirmed a higher risk of IL-1RT1 + LAG3, with and without TNFSF13B + EGLN1‡, to contribute to the prediction of ISM diagnosis against AdvSM (*P* < 0.01) (Table 2).

**Table 2:** Exploratory Binary Logistic Regression Analysis of the Outcome Diagnosis Based on the Top Confirmed Biomarkers

Outcome	Model	C-index	ΔC-index∗	β	SEM	OR	95% CI	*P* value	Combined model	C-index	ΔC-index†	LR‡	*P* value
ISM (vs AdvSM)	IL-1RT1	0.895	0.185	−3.57	0.90	0.03	0.005–0.16	<0.0001					
	LAG3	0.825	0.115	−2.89	0.80	0.06	0.01–0.27	<0.001	IL-1RT1 + LAG3	0.898	0.003	6.87	0.009
	TNFSF13B	0.845	0.135	−1.55	0.47	0.21	0.08–0.54	0.001	IL-1RT1 + LAG3 + TNFSF13B	0.902	0.004	2.07	0.15
	EGLN1§	0.851	0.141	−3.05	1.04	0.05	0.006–0.37	0.004	IL-1RT1 + LAG3 + TNFSF13B + EGLN1§	0.960	0.058	7.04	0.008
	IL-18BP	0.893	0.183	−3.45	0.86	0.03	0.006–0.17	<0.0001	IL-1RT1 + LAG3 + EGLN1§ + IL-18BP	0.959	-0.001	0.38	0.54
ISM (vs PCV)	TPSAB1	0.945	0.193	−0.091	0.023	0.91	0.87–0.96	<0.0001					
	FGF2	0.895	0.143	−1.48	0.29	0.23	0.13–0.40	<0.0001	TPSAB1 + FGF2	0.979	0.033	3.27	<0.001
	MILR1	0.890	0.138	2.12	0.44	8.32	3.52–19.59	<0.0001	TPSAB1 + FGF2 + MILR1	0.988	0.009	10.77	0.001
	TR	0.871	0.119	−1.36	0.28	0.26	0.15–0.44	<0.0701	TPSAB1 + FGF2 + MILR1 + TR	0.993	0.005	8.16	0.004
	DPP10	0.871	0.119	−4.56	0.95	0.01	0.002–0.07	<0.0001	TPSAB1 + FGF2 + MILR1 + TR + DPP10	0.996	0.003	6.09	0.01
	EDAR	0.897	0.14	1.96	0.36	7.08	3.47–14.45	<0.0001	TPSAB1 + FGF2 + MILR1 + TR + DPP10 + EDAR	0.996	0	3.04	0.08
AdvSM (vs PCV)	MILR1	0.886	0.317	1.96	0.525	0.525	2.55–19.94	<0.0001					
	IL-1RT1	0.923	0.354	5.94	1.891	1.891	9.39–15,526.13	0.002	MILR1 + IL-1RT1	0.916	0.03	3.98	0.046
	GRN	0.953	0.384	4.51	1.301	1.301	7.09–1165.43	<0.001	MILR1 + IL-1RT1 + GRN	0.983	0.02	2.48	0.11
	IL2-RA	0.894	0.325	2.20	0.586	0.586	2.87–28.57	<0.001	MILR1 + IL-1RT1 + GRN + IL2-RA	0.942	0.004	0.07	0.80

β, estimate; ΔC index, ΔC statistic; AdvSM, advanced systemic mastocytosis; DPP10, inactive dipeptidyl peptidase 10; EDAR, tumor necrosis factor receptor superfamily member EDAR; EGLN1, Egl nine homolog 1; FGF2, fibroblast growth factor 2; GRN, granulin; IL-1RT1, IL-1 receptor type 1; IL2-RA, IL-2 receptor subunit alpha; IL-18BP, IL-18-binding protein; ISM, indolent systemic mastocytosis; LAG3, lymphocyte activation gene 3; LR, likelihood ratio χ^2^; MILR1, allergin-1; OR, odds ratio; PCV, polycythemia vera; TNFSF13B, tumor necrosis factor ligand superfamily member 13B; TPSAB1, tryptase alpha/beta-1; TR, transferrin receptor 1.

All single models and combined models are adjusted to age (ie, diagnosis ∼ age + marker_i_).

∗ ΔC index, difference in C-index between a model with and without the marker.

† ΔC index, difference in C-index between a model and the same model without the additional marker.

‡ Difference in likelihood ratio between a model and the same model without the additional marker.

§ Limit of detection (LOD), markers with >20% of samples below LOD.

The top biomarkers [TPSAB1, FGF2, MILR1, TR, inactive dipeptidyl peptidase 10 (DPP10), and EDAR] identified by Boruta were confirmed as independent predictors of ISM versus PCV (*P* < 0.0001). Of them, TPSAB1, MILR1, and EDAR had the greatest odds ratios. Step-by-step addition of up to five biomarkers significantly added to the prediction model (*P* < 0.01), in particular the model combining TPSAB1, FGF2, and MILR1 (Table 2). Last, patients with AdvSM could be distinguished from patients with PCV based on MILR1, IL-1RT1, GRN, or IL2-RA, which, individually, contributed to the model significantly (*P* < 0.0001) (Table 2). However, only the model including MILR1 and IL-1RT1 improved the ability of prediction (*P* < 0.0001) (Table 2).

#### Linkage of Relevant Plasma Biomarkers and Single-Cell RNA-Sequencing Data

**Figure 5:**
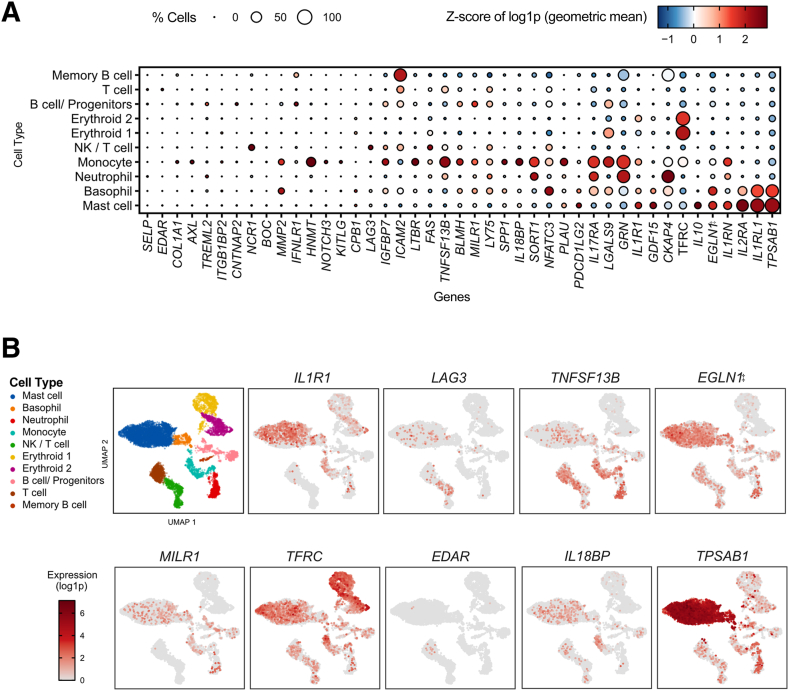
Gene expression analysis of proteins in a single-cell transcriptomic landscape of bone marrow mononuclear cells from patients with indolent systemic mastocytosis (ISM). Annotated single-cell transcriptomics data from three patients with ISM. **A:** Dot plot of the mean gene expression (Z-score; gene-wise standardized) per cell type. The size of the dot is proportional to the percentage of cells that express the corresponding gene. Genes are ordered on the basis of the mean expression values in mast cells. **B:** Uniform manifold approximation and projection (UMAP) of the data set published by Söderlund et al^8^ with refined annotated cell types^34^ and the top nine individual genes (*IL1R1* to *TPSAB1*) associated to ISM at protein expression level. In each separate UMAP, the color code indicates the normalized expression in each individual. Single-cell transcriptomic data set is available at the Gene Expression Omnibus database^8^ (*https://www.ncbi.nlm.nih.gov/geo*; accession number GSE222830). NK, natural killer.

To explore the potential cellular source of the most relevant proteins associated to SM subtypes, an open-access scRNA-seq data set was used.^8^ Considering that such data set was generated from ISM bone marrow mononuclear cells, the present study evaluated the gene expression of only those proteins detected in the predictive analysis to be associated to ISM (Figure 5A and Supplemental Figure S2). A dot plot and uniform manifold approximation and projection showed the gene expression of selected proteins in a single-cell transcriptomic landscape, with detailed annotations of the individual cell populations and gene expression level of the top biomarkers shown in Figure 5B. Mast cells had the greatest gene expression of *TPSAB1* and *IL2RA* (CD25), along with *IL-1RL1* [suppression of tumorigenicity 2 (ST2)] (Figure 5). Notably, *IL10* was detected in a large proportion of mast cells (Figure 5A), whereas *LAG3*, also detectable in mast cells, had greater average expression in natural killer/T cells. *GRN*, on the contrary, was associated with neutrophils and monocytes. *MILR1* gene expression was found in mast cells, basophils, B cells/progenitors, and monocytes. The expression levels of genes, such as *EDAR* and *KITLG* (stem cell factor), were low in all cell populations.

## Discussion

This exploratory study was initiated to investigate the plasma proteome in ISM compared with AdvSM, and how this relates to another myeloproliferative disease (ie, polycythemia vera). Here, this study demonstrated that patients with ISM and AdvSM can be differentiated on the basis of their plasma protein profiles. Furthermore, using patients with PCV as reference, the distinct plasma protein profiles associated to ISM and AdvSM might indicate different underlying disease mechanisms that possibly predict different SM subtypes. Validation of protein expression from patients with ISM in a publicly available scRNA-seq data set demonstrated that the identified biomarkers are potentially expressed in other immune cell types besides mast cells, as well as in structural cells not investigated in the present study.

Beyond serum tryptase,^35^ and urine metabolites of histamine and prostaglandin D_2_,^6^ others have previously explored novel and potential biomarkers to broaden the insight into the pathophysiology of SM.^8^^,^^10^^,^^11^^,^^19^^,^^36^ Along the same line of such exploratory studies, in the present study, 275 unique proteins have been screened in a clinically diverse cohort of mastocytosis that included patients with indolent disease course and patients with unfavorable prognosis (ie, AdvSM). In agreement with previous studies,^8^^,^^10^^,^^11^^,^^19^^,^^36^ this study detected altered levels of different immune-related proteins differing between ISM and more severe cases of SM. In this study, when comparing patients with ISM against patients with AdvSM, plasma proteins related to cardiovascular disease and immune response showed a good separation. Most of the differentiating proteins contributing to this separation were detected in increased plasma levels in the AdvSM group compared with those in the ISM group, suggesting that different molecular mechanisms may explain distinct disease severities. Among the relevant proteins identified in the Boruta model, high expression of MILR1, IL2-RA (CD25), IL-1RT1, and IL10 is in agreement with previous studies that have linked these markers to mast cell burden and/or mastocytosis,^8^^,^^16^^,^^17^^,^^37^ and may therefore be indicative of greater disease activity in patients with AdvSM rather than in patients with ISM. For instance, another study associated high levels of the proinflammatory cytokine IL-1RT1 to patients with ISM reporting a mast cell–dependent flare or a mast cell activation event.^37^ In contrast, higher levels of IL-1RT1 were linked to the clinically severe cases of AdvSM included in this study, being mast cells are the predominant cell type expressing *IL-1R1* at the mRNA level.

LAG3 has above all been studied in the context of a potential immunotherapeutic target for cancer. In agreement with a recently published study,^8^ increased levels of LAG3 in AdvSM, but not in ISM, were found, potentially reflecting a state of T-cell dysfunction in an inflammatory environment. Furthermore, LAG3 expression might explain the high levels of its ligand galectin-3 (Gal-3) expressed by mast cells and involved in T-cell response modulations^38^ and mast cell mediator release.^39^ Our group has previously shown that plasma Gal-3 levels are increased in patients with ISM with anaphylaxis compared with those without anaphylaxis,^16^ whereas this study could not reveal altered levels of Gal-3 in ISM. Like serum IL-18BP level may differentiate patients with urticaria from those experiencing anaphylaxis,^40^ the differences of IL-18BP in plasma might be related to differences in IL-18 neutralization, although plasma IL-18 levels did not seem to differ between the study groups (ISM and AdvSM). Interestingly, the levels of SCF, the most essential growth factor for mast cell biology, were lower in the AdvSM group (versus ISM), which could indicate a larger dysbalance in the signaling cascade of mutated KIT tyrosine-protein kinase (KIT) in patients with AdvSM.^41^

As a reference group, another hematological disease was chosen to exclude plasma protein profiles that might be related to hematological disorders per se. PCV is characterized by overproduction of clonal *JAK2*-mutated erythrocytes in the bone marrow.^21^ Despite evidence of a common mast cell–erythroid differentiation trajectory during mast cell differentiation,^42^ and mast cell mediators participating in PCV-related symptoms, such as pruritus,^25^^,^^26^ there is no evidence of aberrant mast cells in PCV. The low expression of the proteins TPSAB1, MILR1, and IL2-RA in patients with PCV supports the minor contribution of mast cells to PCV pathophysiology. Although there still was a large number of proteins detected in similar levels in PCV and SM subtypes, this study was designed to characterize mastocytosis-specific proteins with potential to explain distinct underlying pathophysiological mechanisms.

When comparing ISM against PCV, the latter demonstrated a more active inflammatory immune response based on the differentiating proteins, led by FGF-2, an inflammatory and angiogenic factor that might be linked to PCV.^43^ Other proteins found to be increased in plasma from patients with PCV, such as TR, may reflect the dysfunctional development of erythrocytes.^44^ Several reports have linked EDAR to cancer,^45^ whereas to our knowledge, there are no previous reports identifying EDAR as a player in the pathophysiology of SM or PCV. The low levels of Gal-9 detected in ISM, and absence in AdvSM, stand in contrast to a previous report where increased Gal-9 expression was associated to AdvSM.^36^ Related to AdvSM, GRN was identified as a potential biomarker and indicator of innate immune response and inflammation in AdvSM pathophysiology.^46^

Despite the fact that mastocytosis is a primary clonal mast cell disorder, other cell populations, like monocytes, plasmacytoid dendritic cells, type 2 innate lymphoid cells, and type 2 helper T cells, can have altered proportions in ISM compared with healthy individuals.^9^^,^^12^ The cellular origin of the proteins of potential interest were not investigated in the patient samples included in this study. However, because of the exploratory nature of the present study and interest to confirm the findings covered in Figures 2 and 3, an open-access scRNA-seq data set generated from ISM bone marrow blood was used. This exploratory analysis supported, for instance, the implication of not only mast cells to express *TNFSF13B* (B-cell activating factor) but also natural killer, B, and T cells. Although nearly exclusive for mast cells, the expression of *TPSAB1*, *IL1R1*, *MILR1*, and *IL10* was also present in other immune cell types.

Although not being the first study exploring potential protein biomarkers in plasma samples in a clinically diverse SM cohort, the main strength of this study study is the coverage of a broad panel of 275 proteins related to immune response and cardiovascular events. The proximity extension assay is an attractive technology to discover candidate biomarkers from a single plasma drop. However, it is based on relative quantification, which limits between-protein and between-study comparisons. Additionally, given the clinical heterogeneity of mastocytosis, the use of untargeted proteomic analysis might improve the identification and insight of additional molecular components contributing to the disorder pathogenesis. The age difference was taken into consideration in the formal analyses, but the influence of other factors, such as group size, heterogeneity of specific diagnoses, concomitant medication, sex, and menstrual cycle phase differences, in the findings cannot be ruled out. The exploratory analyses of Table 2 are likely positively biased in their predictive performance because of small sample size of AdvSM as well as the variable selection in the same data set. Concerning the patients with CM, they were finally excluded from the present exploratory analyses, although the similarities on plasma protein profile and simultaneous divergence on extracutaneous organ involvement compared with patients with ISM highlight the need for further and additional investigations of similar and dissimilar disease mechanisms in future studies. Additionally, given the clinical heterogeneity of mastocytosis, the use of untargeted proteomic analysis might improve the identification and insight of additional molecular components contributing to the disorder pathogenesis. Screening and characterization of novel biomarkers may improve the understanding of the mastocytosis-specific mechanisms of a clinically diverse disease, refining the diagnosis and prognosis, and contribute to the development of new treatments. The shortlisted molecular biomarkers can partly explain the disparate clinical manifestations between mastocytosis subtypes. Furthermore, most of these biomarkers show potential to predict and identify ISM and AdvSM, and they are not exclusively expressed by mast cells. Future studies can help identify if some of these markers may be associated with disease severity, specific symptoms, and distinct molecular mechanisms.

## Disclosure Statement

None declared.

## Supplemental Data

Supplemental Table S4

Supplemental Figure S1 The protein profile of plasma samples of patients with systemic mastocytosis (SM) and polycythemia vera (PCV). Plasma proteins were analyzed using Olink technology. Principal component (PC) analysis score scatterplot based on the plasma protein profile showing patients with SM (light gray circles) and PCV (dark gray triangles). The centroids depict the group arithmetic mean, and the *P* value supports the distance between the study groups' centroids. *n* = 275 detected proteins; *n* = 92 patients with SM; *n* = 60 patients with PCV.

Supplemental Figure S2 Gene expression analysis of proteins in a single-cell transcriptomic landscape of bone marrow plasma mononuclear cells enriched for mast cells in patients with indolent systemic mastocytosis (ISM). Annotated single-cell transcriptomics data from three patients with ISM published by Söderlund et al.^8^ Dot plot of the mean gene expression per cell type. The size of the dot is proportional to the percentage of cells that express the corresponding gene. Genes are ordered on the basis of the mean expression values in mast cells. Single-cell transcriptomic data set is available at the Gene Expression Omnibus database (*https://www.ncbi.nlm.nih.gov/geo*; accession number GSE222830).^8^ NK, natural killer.
