# Supplementary material for: Plasma Protein Profiling to Discern Indolent from Advanced Systemic Mastocytosis
Source: J Mol Diagn. 2024 Jun 24;26(9):792–804. doi: 10.1016/j.jmoldx.2024.05.010 (PMC12178383; doi:10.1016/j.jmoldx.2024.05.010)
Supplement: Supplemental Table S1 [file mmc1.docx]

**Supplemental Table S1.** Frequency of specific clinical diagnosis in the mastocytosis cohort.

|  | CM  (n= 16) |  | ISM  (n=80) |  | AdvSM  (n=12) |
| --- | --- | --- | --- | --- | --- |
| *CM* | 16 | *ISM* | 77 | *ASM* | 4 |
|  |  | *SSM* | 3 | *ASM-AHN (ASM+MPN)* | 2 |
|  |  |  |  | *ISM-AHN (n=6)* |  |
|  |  |  |  | *ISM+MPN* | 2 |
|  |  |  |  | *ISM+MDS* | 1 |
|  |  |  |  | *ISM+CLL* | 1 |
|  |  |  |  | *ISM+CMML* | 1 |
|  |  |  |  | *ISM+ET* | 1 |

CM, Cutaneous Mastocytosis; ISM, Indolent Systemic Mastocytosis; AdvSM, Advanced SM; SSM, Smoldering SM; ASM, Aggressive Systemic Mastocytosis; ASM-AHN, Aggressive Systemic Mastocytosis with an Associated Hematological Neoplasm; ISM-AHN, Indolent Systemic Mastocytosis with an Associated Hematological Neoplasm; CMML, Chronic Myelomonocytic leukemia; CLL, Chronic lymphocytic leukemia; MDS, myelodysplastic syndrome; MPN, myeloproliferative neoplasms; CLL, chronic lymphocytic leukemia; ET, essential thrombocythemia.
