# Supplementary material for: Plasma Protein Profiling to Discern Indolent from Advanced Systemic Mastocytosis
Source: J Mol Diagn. 2024 Jun 24;26(9):792–804. doi: 10.1016/j.jmoldx.2024.05.010 (PMC12178383; doi:10.1016/j.jmoldx.2024.05.010)
Supplement: Supplemental Table S2 [file mmc2.docx]

**Supplemental Table S2**. List of proteins included in the Olink Target 96 Cardiovascular II (v.5006) panel, percentage of samples below limit of level of detection (%) and frequency of missing data.

|  | Abbreviation | Full protein name | <LOD (% of samples) | Missing data frequency |
| --- | --- | --- | --- | --- |
| 1 | ACE2 | Angiotensin-converting enzyme 2 | 0 | 1 |
| 2 | ADAM-TS13 | A disintegrin and metalloproteinase with thrombospondin motifs 13 | 0 | 1 |
| 3 | ADM | ADM | 0 | 1 |
| 4 | AGRP | Agouti-related protein | 0 | 1 |
| 5 | AMBP | Protein AMBP | 0 | 1 |
| 6 | ANGPT1 | Angiopoietin-1 | 0 | 1 |
| 7 | BMP-6 | Bone morphogenetic protein 6 | 0 | 1 |
| 8 | BNP^a^ | Natriuretic peptides B | 21.43 | 1 |
| 9 | BOC | Brother of CDO | 0 | 1 |
| 10 | CA5A^*^ | Carbonic anhydrase 5A, mitochondrial | 42.26 | 1 |
| 11 | CCL17 | C-C motif chemokine 17 | 0 | 1 |
| 12 | CCL3 | C-C motif chemokine 3 | 0 | 1 |
| 13 | CD4 | T-cell surface glycoprotein CD4 | 0 | 1 |
| 14 | CD40-L | CD40 ligand | 0 | 1 |
| 15 | CD84 | SLAM family member 5 | 0 | 1 |
| 16 | CEACAM8 | Carcinoembryonic antigen-related cell adhesion molecule 8 | 0 | 1 |
| 17 | CTRC | Chymotrypsin C | 0 | 1 |
| 18 | CTSL1 | Cathepsin L1 | 0 | 1 |
| 19 | CXCL1 | C-X-C motif chemokine 1 | 0 | 1 |
| 20 | DCN | Decorin | 0 | 1 |
| 21 | DECR1 | 2,4-dienoyl-CoA reductase, mitochondrial | 0 | 1 |
| 22 | Dkk-1 | Dickkopf-related protein 1 | 0 | 1 |
| 23 | FABP2 | Fatty acid-binding protein, intestinal | 0 | 1 |
| 24 | FGF-21 | Fibroblast growth factor 21 | 0 | 1 |
| 25 | FGF-23 | Fibroblast growth factor 23 | 3.57 | 1 |
| 26 | FS | Follistatin | 0 | 1 |
| 27 | Gal-9 | Galectin-9 | 0 | 1 |
| 28 | GDF-2 | Growth/differentiation factor 2 | 0 | 1 |
| 29 | GH | Growth hormone | 0 | 1 |
| 30 | GIF | Gastric intrinsic factor | 0 | 1 |
| 31 | GLO1 | Lactoylglutathione lyase | 0 | 1 |
| 32 | GT | Gastrotropin | 0.59 | 1 |
| 33 | HAOX1 | Hydroxyacid oxidase 1 | 0 | 1 |
| 34 | HB-EGF | Proheparin-binding EGF-like growth factor | 0 | 1 |

Olink Target 96 Cardiovascular II (v.5006) panel continues in next page.

*(Continues)*

|  | Abbreviation | | Protein name | <LOD (% of samples) | Missing data frequency |
| --- | --- | --- | --- | --- | --- |
| 35 | HO-1 | Heme oxygenase 1 | | 0 | 1 |
| 36 | hOSCAR | Osteoclast-associated immunoglobulin-like receptor | | 0 | 1 |
| 37 | HSP 27 | Heat shock 27 kDa protein | | 0 | 1 |
| 38 | IDUA | Alpha-L-iduronidase | | 0 | 1 |
| 39 | IgG Fc receptor II-b | Low affinity immunoglobulin gamma Fc region receptor II-b | | 0 | 1 |
| 40 | IL16 | Pro-interleukin-16 | | 0 | 1 |
| 41 | IL-17D | Interleukin-17D | | 1.19 | 1 |
| 42 | IL18 | Interleukin-18 | | 0 | 1 |
| 43 | IL-1ra | Interleukin-1 receptor antagonist protein | | 0 | 1 |
| 44 | IL1RL2 | Interleukin-1 receptor-like 2 | | 0 | 1 |
| 45 | IL-27 | Interleukin-27 | | 0 | 1 |
| 46 | IL-4RA | Interleukin-4 receptor subunit alpha | | 0 | 1 |
| 47 | IL6 | Interleukin-6 | | 4.76 | 1 |
| 48 | ITGB1BP2 | Melusin | | 0 | 1 |
| 49 | KIM1 | Kidney Injury Molecule | | 0 | 1 |
| 50 | LEP | Leptin | | 0.60 | 1 |
| 51 | LOX-1 | Lectin-like oxidized LDL receptor 1 | | 0 | 1 |
| 52 | LPL | Lipoprotein lipase | | 0 | 1 |
| 53 | MARCO | Macrophage receptor MARCO | | 0 | 1 |
| 54 | MERTK | Tyrosine-protein kinase Mer | | 0 | 1 |
| 55 | MMP12 | Matrix metalloproteinase-12 | | 0 | 1 |
| 56 | MMP7 | Matrix metalloproteinase-7 | | 0 | 1 |
| 57 | NEMO | NF-kappa-B essential modulator | | 0 | 1 |
| 58 | PAPPA | Pappalysin-1 | | 0 | 1 |
| 59 | PAR-1 | Proteinase-activated receptor 1 | | 0 | 1 |
| 60 | PARP-1 | Poly [ADP-ribose] polymerase 1 | | 0 | 1 |
| 61 | PDGF subunit B | Platelet-derived growth factor subunit B | | 0 | 1 |
| 62 | PD-L2 | Programmed cell death 1 ligand 2 | | 0 | 1 |
| 63 | PGF | Placenta growth factor | | 0 | 1 |
| 64 | PIgR | Polymeric immunoglobulin receptor | | 0 | 1 |
| 65 | PRELP | Prolargin | | 0 | 1 |
| 66 | PRSS27 | Serine protease 27 | | 0 | 1 |
| 67 | PRSS8 | Prostasin | | 0 | 1 |
| 68 | PSGL-1 | P-selectin glycoprotein ligand 1 | | 0 | 1 |
| 69 | PTX3 | Pentraxin-related protein PTX3 | | 0 | 1 |
| 70 | RAGE | Receptor for advanced glycosylation end products | | 0 | 1 |
| 71 | REN | Renin | | 0 | 1 |

Olink Target 96 Cardiovascular II (v.5006) continues in next page.

*(Continues)*

|  | Abbreviation | Protein name | <LOD (% of samples) | Missing data frequency |
| --- | --- | --- | --- | --- |
| 72 | SCF | Stem cell factor | 0 | 1 |
| 73 | SERPINA12 | Serpin A12 | 0 | 1 |
| 74 | SLAMF7 | SLAM family member 7 | 11.90 | 1 |
| 75 | SOD2 | Superoxide dismutase [Mn], mitochondrial | 0 | 1 |
| 76 | SORT1 | Sortilin | 0 | 1 |
| 77 | SPON2 | Spondin-2 | 0 | 1 |
| 78 | SRC | Proto-oncogene tyrosine-protein kinase Src | 0 | 1 |
| 79 | STK4 | Serine/threonine-protein kinase 4 | 0 | 1 |
| 80 | TF | Tissue factor | 0 | 1 |
| 81 | TGM2 | Protein-glutamine gamma-glutamyltransferase 2 | 0 | 1 |
| 82 | THBS2 | Thrombospondin-2 | 0 | 1 |
| 83 | THPO | Thrombopoietin | 0 | 1 |
| 84 | TIE2 | Angiopoietin-1 receptor | 0 | 1 |
| 85 | TM | Thrombomodulin | 0 | 1 |
| 86 | TNFRSF10A | Tumor necrosis factor receptor superfamily member 10A | 0 | 1 |
| 87 | TNFRSF11A | Tumor necrosis factor receptor superfamily member 11A | 0 | 1 |
| 88 | TNFRSF13B | Tumor necrosis factor receptor superfamily member 13B | 0 | 1 |
| 89 | TRAIL-R2 | TNF-related apoptosis-inducing ligand receptor 2 | 0 | 1 |
| 90 | VEGFD | Vascular endothelial growth factor D | 0 | 1 |
| 91 | VSIG2 | V-set and immunoglobulin domain-containing protein 2 | 0 | 1 |
| 92 | XCL1 | C-X-C motif chemokine 1 | 0 | 1 |

LOD, Limit of Detection. ^*^Markers with more than 20% of samples below LOD.
