# Supplementary material for: Plasma Protein Profiling to Discern Indolent from Advanced Systemic Mastocytosis
Source: J Mol Diagn. 2024 Jun 24;26(9):792–804. doi: 10.1016/j.jmoldx.2024.05.010 (PMC12178383; doi:10.1016/j.jmoldx.2024.05.010)
Supplement: Supplemental Table S3 [file mmc3.docx]

**Supplemental Table S3.** List of proteins included in the Olink Target 96 Cardiovascular III (v.6113) panel, percentage of samples below limit of level of detection (%) and frequency of missing data.

|  | Abbreviation | Protein name | <LOD (% of samples) | Missing data frequency |
| --- | --- | --- | --- | --- |
| 1 | ALCAM | CD166 antigen | 0 | 0 |
| 2 | AP-N | Aminopeptidase N | 0 | 0 |
| 3 | AXL | Tyrosine-protein kinase receptor UFO | 0 | 0 |
| 4 | AZU1 | Azurocidin | 0 | 0 |
| 5 | BLM hydrolase | Bleomycin hydrolase | 0 | 0 |
| 6 | CASP-3 | Caspase-3 | 0 | 0 |
| 7 | CCL15 | C-C motif chemokine 15 | 0 | 0 |
| 8 | CCL16 | C-C motif chemokine 16 | 0 | 0 |
| 9 | CCL24 | C-C motif chemokine 24 | 0 | 0 |
| 10 | CD163 | Scavenger receptor cysteine-rich type 1 protein M130 | 0 | 0 |
| 11 | CD93 | Complement component C1q receptor | 0 | 0 |
| 12 | CDH5 | Cadherin-5 | 0 | 0 |
| 13 | CHI3L1 | Chitinase-3-like protein 1 | 0 | 0 |
| 14 | CHIT1 | Chitotriosidase-1 | 2.38 | 0 |
| 15 | CNTN1 | Contactin-1 | 0 | 0 |
| 16 | COL1A1 | Collagen alpha-1(I) chain | 0 | 0 |
| 17 | CPA1 | Carboxypeptidase A1 | 0 | 0 |
| 18 | CPB1 | Carboxypeptidase B | 0 | 0 |
| 19 | CSTB | Cystatin-B | 0 | 0 |
| 20 | CTSD | Cathepsin D | 0 | 0 |
| 21 | CTSZ | Cathepsin Z | 0 | 0 |
| 22 | CXCL16 | C-X-C motif chemokine 16 | 0 | 0 |
| 23 | DLK-1 | Protein delta homolog 1 | 0 | 0 |
| 24 | EGFR | Epidermal growth factor receptor | 0 | 0 |
| 25 | Ep-CAM | Epithelial cell adhesion molecule | 0 | 0 |
| 26 | EPHB4 | Ephrin type-B receptor 4 | 0 | 0 |
| 27 | FABP4 | Fatty acid-binding protein, adipocyte | 0 | 0 |
| 28 | FAS | Tumor necrosis factor receptor superfamily member 6 | 0 | 0 |
| 29 | Gal-3 | Galectin-3 | 0 | 0 |
| 30 | Gal-4 | Galectin-4 | 0 | 0 |
| 31 | GDF-15 | Growth/differentiation factor 15 | 0 | 0 |
| 32 | GP6 | Platelet glycoprotein VI | 0 | 0 |
| 33 | GRN | Granulins | 0 | 0 |
| 34 | ICAM-2 | Intercellular adhesion molecule 2 | 0 | 0 |
| 35 | IGFBP-1 | Insulin-like growth factor-binding protein 1 | 1.19 | 0 |
| 36 | IGFBP-2 | Insulin-like growth factor-binding protein 2 | 0 | 0 |

Olink Target 96 Cardiovascular III (v.6113) continues in next page.

*(Continues)*

|  | Abbreviation | Protein name | <LOD (% of samples) | Missing data frequency |
| --- | --- | --- | --- | --- |
| 37 | IGFBP-7 | Insulin-like growth factor-binding protein 7 | 0 | 0 |
| 38 | IL-17RA | Interleukin-17 receptor A | 0 | 0 |
| 39 | IL-18BP | Interleukin-18-binding protein | 0 | 0 |
| 40 | IL-1RT1 | Interleukin-1 receptor type 1 | 0 | 0 |
| 41 | IL-1RT2 | Interleukin-1 receptor type 2 | 0 | 0 |
| 42 | IL2-RA | Interleukin-2 receptor subunit alpha | 0 | 0 |
| 43 | IL-6RA | Interleukin-6 receptor subunit alpha | 0 | 0 |
| 44 | ITGB2 | Integrin beta-2 | 0 | 0 |
| 45 | JAM-A | Junctional adhesion molecule A | 0 | 0 |
| 46 | KLK6 | Kallikrein-6 | 0 | 0 |
| 47 | LDL receptor | Low-density lipoprotein receptor | 0 | 0 |
| 48 | LTBR | Lymphotoxin-beta receptor | 0 | 0 |
| 49 | MB | Myoglobin | 0 | 0 |
| 50 | MCP-1 | Monocyte chemotactic protein 1 | 0 | 0 |
| 51 | MEPE | Matrix extracellular phosphoglycoprotein | 0 | 0 |
| 52 | MMP-2 | Matrix metalloproteinase-2 | 0 | 0 |
| 53 | MMP-3 | Matrix metalloproteinase-3 | 0 | 0 |
| 54 | MMP-9 | Matrix metalloproteinase-9 | 0 | 0 |
| 55 | MPO | Myeloperoxidase | 0 | 0 |
| 56 | Notch 3 | Neurogenic locus notch homolog protein 3 | 0 | 0 |
| 57 | NT-proBNP | N-terminal prohormone brain natriuretic peptide | 1.19 | 0 |
| 58 | OPG | Osteoprotegerin | 0 | 0 |
| 59 | OPN | Osteopontin | 0 | 0 |
| 60 | PAI | Plasminogen activator inhibitor 1 | 0 | 0 |
| 61 | PCSK9 | Proprotein convertase subtilisin/kexin type 9 | 0 | 0 |
| 62 | PDGF subunit A | Platelet-derived growth factor subunit A | 0 | 0 |
| 63 | PECAM-1 | Platelet endothelial cell adhesion molecule | 0 | 0 |
| 64 | PGLYRP1 | Peptidoglycan recognition protein 1 | 0 | 0 |
| 65 | PI3 | Elafin | 0 | 0 |
| 66 | PLC | Perlecan | 0 | 0 |
| 67 | PON3 | Paraoxonase | 0 | 0 |
| 68 | PRTN3 | Myeloblastin | 0 | 0 |

|  | Abbreviation | Protein name | <LOD (% of samples) | Missing data frequency |
| --- | --- | --- | --- | --- |
| 69 | PSP-D | Pulmonary surfactant-associated protein D | 11.90 | 0 |
| 70 | RARRES2 | Retinoic acid receptor responder protein 2 | 0 | 0 |
| 71 | RETN | Resistin | 0 | 0 |
| 72 | SCGB3A2 | Secretoglobin family 3A member 2 | 0 | 0 |
| 73 | SELE | E-selectin | 0 | 0 |
| 74 | SELP | P-selectin | 0 | 0 |
| 75 | SHPS-1 | Tyrosine-protein phosphatase non-receptor type substrate 1 | 0 | 0 |
| 76 | SPON1^*^ | Spondin-1 | 86.90 | 0 |
| 77 | ST2 | ST2 protein | 0 | 0 |
| 78 | TFF3 | Trefoil factor 3 | 0 | 0 |
| 79 | TFPI | Tissue factor pathway inhibitor | 0 | 0 |
| 80 | TIMP4 | Metalloproteinase inhibitor 4 | 0 | 0 |
| 81 | TLT-2 | Trem-like transcript 2 protein | 0 | 0 |
| 82 | TNF-R1 | Tumor necrosis factor receptor 1 | 0 | 0 |
| 83 | TNF-R2 | Tumor necrosis factor receptor 2 | 0 | 0 |
| 84 | TNFRSF10C | Tumor necrosis factor receptor superfamily member 10C | 0.60 | 0 |
| 85 | TNFRSF14 | Tumor necrosis factor receptor superfamily member 14 | 0 | 0 |
| 86 | TNFSF13B | Tumor necrosis factor ligand superfamily member 13B | 0 | 0 |
| 87 | t-PA | Tissue-type plasminogen activator | 0 | 0 |
| 88 | TR | Transferrin receptor protein 1 | 0 | 0 |
| 89 | TR-AP | Tartrate-resistant acid phosphatase type 5 | 0 | 0 |
| 90 | uPA | Urokinase-type plasminogen activator | 0 | 0 |
| 91 | U-PAR | Urokinase plasminogen activator surface receptor | 0 | 0 |
| 92 | vWF | von Willebrand factor | 0 | 0 |

LOD, Limit of Detection. ^*^Markers with more than 20% of samples below LOD.
