# Supplementary material for: Plasma Protein Profiling to Discern Indolent from Advanced Systemic Mastocytosis
Source: J Mol Diagn. 2024 Jun 24;26(9):792–804. doi: 10.1016/j.jmoldx.2024.05.010 (PMC12178383; doi:10.1016/j.jmoldx.2024.05.010)
Supplement: Supplemental Table S4 [file mmc4.docx]

**Supplemental Table S4.** List of proteins included in the Olink Target 96 Immune response (v. 3204) panel, percentage of samples below limit of level of detection (%) and frequency of missing data.

|  | Abbreviation | Protein name | <LOD (% of samples) | Missing data frequency |
| --- | --- | --- | --- | --- |
| 1 | AREG | Amphiregulin (AR) | 0 | 0 |
| 2 | ARNT^*^ | Aryl hydrocarbon receptor nuclear translocator | 86.31 | 0 |
| 3 | BACH1 | Transcription regulator protein BACH1 | 0 | 0 |
| 4 | BIRC2^*^ | Baculoviral IAP repeat-containing protein 2 | 56.55 | 0 |
| 5 | BTN3A2 | Butyrophilin subfamily 3 member A2 | 0 | 0 |
| 6 | CCL11 | Eotaxin | 0 | 0 |
| 7 | CD28^*^ | T-cell-specific surface glycoprotein CD28 | 79.76 | 0 |
| 8 | CD83 | CD83 antigen | 0 | 0 |
| 9 | CDSN | Corneodesmosin | 0 | 0 |
| 10 | CKAP4 | Cytoskeleton-associated protein 4 | 0 | 0 |
| 11 | CLEC4A | C-type lectin domain family 4 member A | 0 | 0 |
| 12 | CLEC4C | C-type lectin domain family 4 member C | 0 | 0 |
| 13 | CLEC4D | C-type lectin domain family 4 member D | 0 | 0 |
| 14 | CLEC4G | C-type lectin domain family 4 member G | 0 | 0 |
| 15 | CLEC6A | C-type lectin domain family 6 member A | 13.10 | 0 |
| 16 | CLEC7A | C-type lectin domain family 7 member A | 1.19 | 0 |
| 17 | CNTNAP2 | Contactin-associated protein-like 2 | 0 | 0 |
| 18 | CXADR | Coxsackievirus and adenovirus receptor | 0 | 0 |
| 19 | CXCL12^*^ | Stromal cell-derived factor 1 | 35.71 | 0 |
| 20 | DAPP1 | Dual adapter for phosphotyrosine and 3-phosphotyrosine and 3-phosphoinositide | 0 | 0 |
| 21 | DCBLD2 | Discoidin, CUB and LCCL domain-containing protein 2 | 0 | 0 |
| 22 | DCTN1 | Dynactin subunit 1 | 0 | 0 |
| 23 | DDX58 | Probable ATP-dependent RNA helicase DDX58 | 0 | 0 |
| 24 | DFFA | DNA fragmentation factor subunit alpha | 0 | 0 |
| 25 | DGKZ^*^ | Diacylglycerol kinase zeta | 74.40 | 0 |
| 26 | DPP10 | Inactive dipeptidyl peptidase 10 | 0.60 | 0 |
| 27 | EDAR | Tumor necrosis factor receptor superfamily member EDAR | 1.19 | 0 |
| 28 | EGLN1^*^ | Egl nine homolog 1 | 76.19 | 0 |
| 29 | EIF4G1 | Eukaryotic translation initiation factor 4 gamma 1 | 0 | 0 |
| 30 | EIF5A^*^ | Eukaryotic translation initiation factor 5A-1 | 89.88 | 0 |
| 31 | FAM3B | Protein FAM3B | 0 | 0 |
| 32 | FCRL3 | Fc receptor-like protein 3 | 9.52 | 0 |
| 33 | FCRL6 | Fc receptor-like protein 6 | 0 | 0 |

Olink Target 96 Immune response (v. 3204) continues in next page.

*(Continues)*

|  | Abbreviation | Protein name | <LOD (% of samples) | Missing data frequency |
| --- | --- | --- | --- | --- |
| 34 | FGF2 | Fibroblast growth factor 2 | 1.19 | 0 |
| 35 | FXYD5 | FXYD domain-containing ion transport regulator 5 | 8.33 | 0 |
| 36 | GALNT3^*^ | Polypeptide N-acetylgalactosaminyltransferase 3 | 41.67 | 0 |
| 37 | GLB1 | Beta-galactosidase | 0 | 0 |
| 38 | HCLS1 | Hematopoietic lineage cell-specific protein | 0 | 0 |
| 39 | HEXIM1 | Protein HEXIM1 | 0 | 0 |
| 40 | HNMT | Histamine N-methyltransferase | 0 | 0 |
| 41 | HSD11B1 | Corticosteroid 11-beta-dehydrogenase isozyme 1 | 0 | 0 |
| 42 | ICA1 | Islet cell autoantigen 1 | 16.07 | 0 |
| 43 | IFNLR1 | Interferon lambda receptor 1 | 0 | 0 |
| 44 | IL10 | Interleukin-10 | 0 | 0 |
| 45 | IL12RB1^*^ | Interleukin-12 receptor subunit beta-1 | 45.83 | 0 |
| 46 | IL5^*^ | Interleukin-5 | 73.81 | 0 |
| 47 | IL6^†^ | Interleukin-6 | 10.71 | 0 |
| 48 | IRAK1 | Interleukin-1 receptor-associated kinase 1 | 0 | 0 |
| 49 | IRAK4 | Interleukin-1 receptor-associated kinase 4 | 0.60 | 0 |
| 50 | IRF9 | Interferon regulatory factor 9 | 1.19 | 0 |
| 51 | ITGA11 | Integrin alpha-11 | 2.38 | 0 |
| 52 | ITGA6 | Integrin alpha-6 | 0 | 0 |
| 53 | ITGB6 | Integrin beta-6 | 0 | 0 |
| 54 | ITM2A | Integral membrane protein 2A | 0 | 0 |
| 55 | JUN^*^ | Transcription factor AP-1 | 61.90 | 0 |
| 56 | KLRD1 | Natural killer cells antigen CD94 | 0 | 0 |
| 57 | KPNA1^*^ | Importin subunit alpha-5 | 80.95 | 0 |
| 58 | KRT19 | Keratin, type I cytoskeletal 19 | 0 | 0 |
| 59 | LAG3 | Lymphocyte activation gene 3 protein | 0 | 0 |
| 60 | LAMP3 | Lysosome-associated membrane glycoprotein 3 | 0 | 0 |
| 61 | LILRB4 | Leukocyte immunoglobulin-like receptor subfamily B member 4 | 0 | 0 |
| 62 | LY75 | Lymphocyte antigen 75 | 0 | 0 |
| 63 | MASP1 | Mannan-binding lectin serine protease 1 | 0 | 0 |
| 64 | MGMT | Methylated-DNA--protein-cysteine methyltransferase | 0 | 0 |
| 65 | MILR1 | Allergin-1 | 0 | 0 |
| 66 | NCR1 | Natural cytotoxicity triggering receptor 1 | 0 | 0 |
| 67 | NF2 | Merlin | 5.95 | 0 |

Olink Target 96 Immune response (v. 3204) continues in next page.

*(Continues)*

|  | Abbreviation | Protein name | <LOD (% of samples) | Missing data frequency |
| --- | --- | --- | --- | --- |
| 68 | NFATC3 | Nuclear factor of activated T-cells, cytoplasmic 3 | 5.36 | 0 |
| 69 | NTF4 | Neurotrophin-4 | 0 | 0 |
| 70 | PADI2^*^ | Protein-arginine deiminase type-2 | 64.29 | 0 |
| 71 | PIK3AP1 | Phosphoinositide 3-kinase adapter protein 1 | 0 | 0 |
| 72 | PLXNA4 | Plexin-A4 | 0 | 0 |
| 73 | PPP1R9B | Neurabin-2 | 0 | 0 |
| 74 | PRDX1 | Peroxiredoxin-1 | 0 | 0 |
| 75 | PRDX3 | Thioredoxin-dependent peroxide reductase, mitochondrial | 7.74 | 0 |
| 76 | PRDX5 | Peroxiredoxin-5, mitochondrial | 0 | 0 |
| 77 | PRKCQ | Protein kinase C theta type | 14.29 | 0 |
| 78 | PSIP1 | PC4 and SFRS1-interacting protein | 0.60 | 0 |
| 79 | PTH1R | Parathyroid hormone/parathyroid hormone-related peptide receptor | 0 | 0 |
| 80 | SH2D1A | SH2 domain-containing protein 1A | 0 | 0 |
| 81 | SIT1 | Signaling threshold-regulating transmembrane adapter 1 | 0 | 0 |
| 82 | SH2B3 | SH2B adapter protein 3 | 0 | 0 |
| 83 | SPRY2 | Protein sprouty homolog 2 | 0.60 | 0 |
| 84 | SRPK2 | SRSF protein kinase 2 | 0 | 0 |
| 85 | STC1 | Stanniocalcin-1 | 0 | 0 |
| 86 | TANK | TRAF family member-associated NF-kappa-B activator | 7.14 | 0 |
| 87 | TPSAB1 | Tryptase alpha/beta-1 | 0 | 0 |
| 88 | TRAF2 | NF receptor-associated factor 2 | 0.60 | 0 |
| 89 | TREM1^*^ | Triggering receptor expressed on myeloid cells 1 | 100 | 0 |
| 90 | TRIM21 | E3 ubiquitin-protein ligase TRIM21 | 0.60 | 0 |
| 91 | TRIM5 | Tripartite motif-containing protein 5 | 0 | 0 |
| 92 | ZBTB16 | Zinc finger and BTB domain-containing protein 16 | 1.19 | 0 |

LOD, Limit of detection. ^*^Markers with more than 20% of samples below LOD. ^†^IL-6 excluded from Olink Target 96 Immune response.
