# Supplementary figures and images for: Plasma Protein Profiling to Discern Indolent from Advanced Systemic Mastocytosis
Source: J Mol Diagn. 2024 Jun 24;26(9):792–804. doi: 10.1016/j.jmoldx.2024.05.010 (PMC12178383; doi:10.1016/j.jmoldx.2024.05.010)

**Supplemental Figure S1**

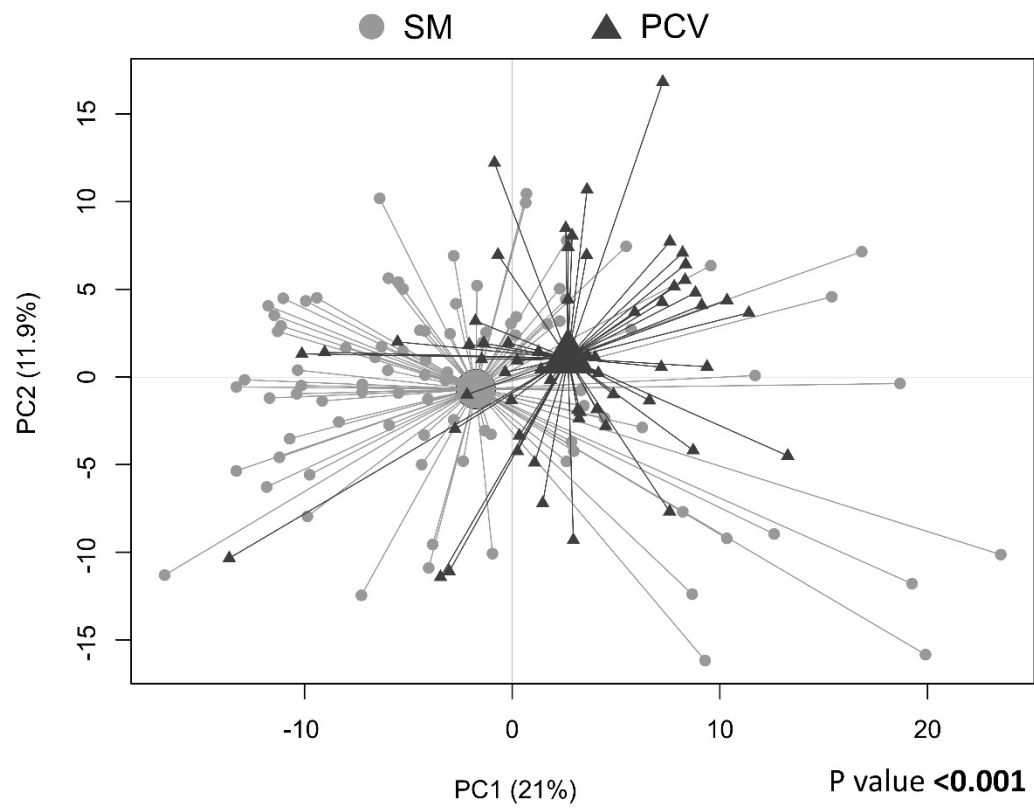

Supplement: Supplemental Figure S1 — The protein profile of plasma samples of patients with systemic mastocytosis (SM) and polycythemia vera (PCV). Plasma proteins were analyzed using Olink technology. Principal component (PC) analysis score scatterplot based on the plasma protein profile showing patients with SM (light gray circles) and PCV (dark gray triangles). The centroids depict the group arithmetic mean, and the P value supports the distance between the study groups' centroids. n = 275 detected proteins; n = 92 patients with SM; n = 60 patients with PCV. [file mmc5.pdf]

## Supplemental Figure S2

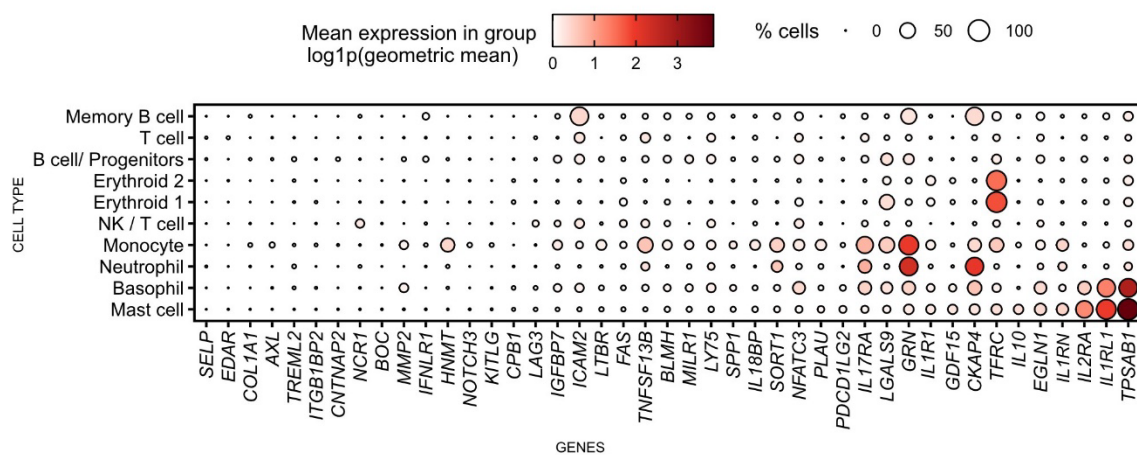

Supplement: Supplemental Figure S2 — Gene expression analysis of proteins in a single-cell transcriptomic landscape of bone marrow plasma mononuclear cells enriched for mast cells in patients with indolent systemic mastocytosis (ISM). Annotated single-cell transcriptomics data from three patients with ISM published by Söderlund et al.8 Dot plot of the mean gene expression per cell type. The size of the dot is proportional to the percentage of cells that express the corresponding gene. Genes are ordered on the basis of the mean expression values in mast cells. Single-cell transcriptomic data set is available at the Gene Expression Omnibus database (https://www.ncbi.nlm.nih.gov/geo; accession number GSE222830).8 NK, natural killer. [file mmc6.pdf]
